# A Role for Notch Signalling in Breast Cancer and Endocrine Resistance

**DOI:** 10.1155/2016/2498764

**Published:** 2016-01-10

**Authors:** Ahmet Acar, Bruno M. Simões, Robert B. Clarke, Keith Brennan

**Affiliations:** ^1^Faculty of Life Sciences, University of Manchester, Oxford Road, Manchester M13 9PT, UK; ^2^Breast Biology Group, Institute of Cancer Sciences, University of Manchester, Wilmslow Road, Manchester M20 4BX, UK; ^3^Breast Cancer Now Research Unit, Institute of Cancer Sciences, University of Manchester, Wilmslow Road, Manchester M20 4BX, UK

## Abstract

Over the past decade, there has been growing interest in the Notch signalling pathway within the breast cancer field. This interest stemmed initially from the observation that Notch signalling is aberrantly activated in breast cancer and its effects on various cellular processes including proliferation, apoptosis, and cancer stem cell activity. However more recently, elevated Notch signalling has been correlated with therapy resistance in oestrogen receptor-positive breast cancer. As a result, inhibiting Notch signalling with therapeutic agents is being explored as a promising treatment option for breast cancer patients.

## 1. The Notch Signalling Pathway

**Figure 1:**
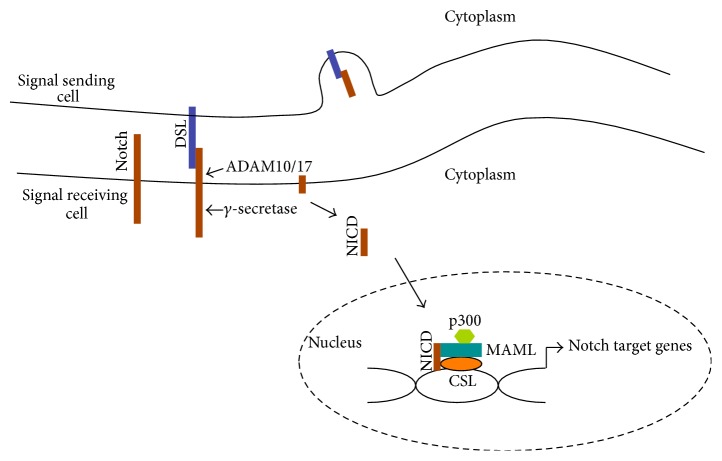
Basics of Notch signalling pathway. Notch signalling activation occurs via an interaction between DSL ligand and Notch receptor on adjacent cells. This interaction leads to force being applied to the extracellular domain of Notch, as DSL ligand undergoes endocytosis into the signalling cell, leading to a conformational change within the negative regulatory region (NRR). This conformational change exposes a cleavage site (S2) for the ADAM10 and ADAM17 proteases. The extracellularly truncated Notch protein then undergoes cleavage at site 3 (S3), mediated by *γ*-secretase, which releases the Notch intracellular domain (NICD). Finally, NICD translocates into the nucleus and interacts with the DNA binding protein RBPj and the transcriptional coactivators MAML and p300 to initiate transcription of downstream targets including the* Hes* and* Hey* family of genes.

The Notch signalling pathway is evolutionarily conserved and is found in organisms as diverse as hydra and humans. In mammals, there are four Notch receptors (Notch1 to Notch4) and five DSL ligands (Jagged1, Jagged2, Delta-like1 (Dll1), Dll3, and Dll4) [1]. As the DSL ligands are transmembrane proteins, Notch signalling is initiated by the interaction of DSL ligands and Notch receptors on adjacent cells. Their interaction leads to a proteolytic cleavage of the Notch protein at the S2 cleavage site mediated by ADAM10 and ADAM17. Following this cleavage, the remaining part of the Notch protein undergoes another proteolytic cleavage mediated by the *γ*-secretase enzyme complex. The latter cleavage releases the Notch intracellular domain (NICD), which translocates to the nucleus. Within the nucleus, NICD forms a complex with the DNA binding protein RBPj and a member of the Mastermind-like (MAML) family transcriptional coactivators (Figure 1). This complex of proteins then activates the transcription of Notch target genes, including members of* Hes* and* Hey* family of transcription. Other target genes include several genes that are directly associated with tumourigenesis such as* cyclinD1* and* Slug* [2, 3]. Notch signalling activity is also regulated through posttranslational modification. For example, Notch receptors are glycosylated through the sequential action of peptide-O-fucosyltransferase (POFUT1) [4] and the Fringe GlcNAc transferases [5]; this modification alters the affinity of Notch for Delta and Serrate/Jagged ligands. Notch proteins are also subject to phosphorylation by glycogen synthase kinase 3*β* (GSK3*β*) [6] and ubiquitination by the E3 ubiquitin ligase FBXW7 [7]. These latter modifications alter the stability of NICD and thereby control the duration of signalling.

## 2. Notch Signalling Is Aberrantly Activated in Breast Cancer

Breast cancer is one of the leading causes of death seen in the world despite the fact that there are ongoing efforts to improve detection and treatment. Breast cancer is a heterogeneous disease and currently is split into three subtypes clinically based on immunohistochemical analysis: ER*α* +ve (oestrogen receptor *α*-positive), HER2 +ve (human epidermal growth factor receptor 2-positive), and triple negative (cancers that lack expression of ER*α*, progesterone receptor (PR), and HER2). Currently, targeted therapies are available for ER*α* and Her2 +ve tumours. In addition to traditional subtype analysis, breast cancer can also be subtyped based on gene expression profiles. Perou and colleagues reported the presence of luminal-like, normal-like, basal-like, and Her2-enriched subtypes [8]. They also split the luminal-like subtype into luminal A and luminal B subtypes. Although not perfectly aligned, the luminal-like, Her2-enriched, and basal-like subtypes correlate with ER*α* +ve, HER2 +ve, and triple negative breast cancer subtypes identified by immunohistochemistry. More recently a sixth molecular subtype has been recognised, the claudin-low subtype [9]. Alternative molecular subtypes that link to copy number aberration data have also been proposed [10].

Over the past decade, aberrant activation of Notch signalling in breast cancer has been reported by many different groups. In invasive breast cancer, the elevated expression of Notch signalling pathway components has been reported, including Jagged1-2, Dll1, Dll3, and Dll4, Notch receptors, and* Hes* and* Hey* target genes [11]. For example, elevated expression of Jagged1 and Notch1 has been linked to poor prognosis in breast cancer patients [12–14]. Likewise, Numb, a negative regulator of Notch signalling, has been found to be lost in 50% of breast cancer through ubiquitination and proteasomal degradation [15]. The accumulation of NICD has also been reported [16].

There are various examples of elevated Notch signalling being associated with a particular subtype of breast cancer and response to targeted therapy. For example, Notch signalling has been shown to be activated in ER*α* +ve breast cancer in response to treatment [17, 18]. Elevated Notch1 expression is also found in HER2 +ve [19] and triple negative/basal breast tumours [13]. In contrast, elevated Notch2 is associated with highly differentiated and poorly proliferative breast cancers [20]. In fact, Notch signalling activation has even been observed in preinvasive breast lesions, including usual ductal hyperplasia (UDH) and ductal carcinoma* in situ* (DCIS) [11, 19, 21]. Lastly, a very recent study has identified a series of activating mutations within the PEST domain of Notch1, Notch2, and Notch3 [22]. These mutations were enriched in triple negative breast cancers.

The tumour promoting activity of Notch is evident from transgenic mouse model studies. For example, Notch4 intracellular domain when expressed under the control of the whey acidic promoter (WAP) or Notch1, Notch3, or Notch4 intracellular domains under the control of the mouse mammary tumour virus (MMTV) promoter all cause tumour formation in mice [23–26]. Also, conditional deletion of Lunatic Fringe in the mammary gland leads to elevated Jagged1-induced Notch signalling and formation of basal-like mammary tumours [27].

## 3. Notch Signalling Regulates Many Cellular Properties of Breast Cancer

Notch signalling is known to regulate many cellular processes including proliferation [17, 28], apoptosis [16, 29], angiogenesis [30, 31], hypoxia [32], cancer stem cell activity [21, 33, 34], epithelial-to-mesenchymal transition (EMT) [3], and metastasis [35]. Notch signalling promotes proliferation in breast cancer cell lines by upregulating* cyclinA*,* cyclinB,* and* cyclinD1* expression [2, 17, 36]. Notch protects breast epithelial cells from apoptosis by activating Akt [16, 29, 37]. It is thought that activation of Akt by Notch in this context occurs through downregulation of PTEN expression or secretion of an autocrine signalling protein [29, 36]. Jagged1, expressed in tumour cells, has been shown to activate Notch signalling in neighbouring endothelial cells to promote angiogenesis [30]. Notch signalling can also regulate angiogenesis by limiting the number of tip cells formed and by promoting the arterial cell fate [31]. Notch signalling also regulates the self-renewal of breast cancer stem cells [21, 34]. In this role, Notch4 appears to be particularly important, as knockdown of Notch4 has a much more significant effect on breast cancer stem cell numbers than Notch1 knockdown [34]. Notch activity induces EMT by means of RBPj binding to promoter sequences upstream of the* Slug* gene [3]. Lastly, Notch activity has been shown to induce metastasis of breast cancer cells to the bone [35]. In this elegant study, the breast cancer cell line MDA-MB-231 was forced to express the Notch ligand Jagged1, and this was found to significantly increase bone metastasis [35]. Altogether, it is very clear that activation of Notch plays a key role in breast cancer. Therefore it represents a very attractive therapeutic target.

## 4. Endocrine Therapy and Targeting Notch in Breast Cancer

Current breast cancer treatments include general chemotherapeutic drugs such as epirubicin, doxorubicin, paclitaxel, and docetaxel, each aiming at blocking the proliferation of breast cancer cells. On the other hand, targeted therapies aim to disrupt the function of a protein or pathway known to drive tumour growth. Three out of four breast cancers express the oestrogen receptor (ER) alpha, and most of these depend on oestrogen for growth [38]. Consequently, targeting the function of ER is one of the most effective approaches to treat breast cancer [39]. Therapies against ER*α* +ve breast cancer include antioestrogens and aromatase inhibitors (AIs) [40]. Tamoxifen, which antagonises the action of ER by competing for the ligand-binding domain of the receptor, is the prevalent endocrine therapy of choice [41]. Other antioestrogens, such as pure antioestrogens or AIs, are considered to be a good choice when tamoxifen treatment fails [42]. AIs are also used in postmenopausal women without ovarian function and act by blocking the synthesis of oestrogen from androgens in the peripheral tissues [43]. Pure antioestrogens, like fulvestrant, block dimerization of the receptor and lead to ubiquitination and proteasome-mediated degradation of the receptor [44].

Unfortunately, ER-positive tumours frequently develop resistance to endocrine treatments and relapse. Although downregulation of ER expression can contribute to endocrine resistance, loss of the receptor during the acquisition of resistance is not commonly observed. Several recent studies have suggested that ER mutations, such as a tyrosine to asparagine substitution at residue 537 (Y537N), may occur during endocrine treatment [45, 46]. These mutations activate ER and as a consequence lead to a loss of endocrine therapy responsiveness. The overexpression of different growth factor receptors and the activation of their downstream signalling pathways are also involved in the acquisition of endocrine resistance. Tumours resistant to tamoxifen are often associated with high expression and enhanced activity of tyrosine kinase receptors EGFR and HER2 [47]. These receptors activate kinases, such as ERK1/2, PI3K, and AKT, which promote phosphorylation and ligand independent activation of ER [38].

Over the past few years, there is accumulating evidence to suggest that cells with stem cell properties are the main driving force of tumourigenesis. Al-Hajj and colleagues were the first to show that a subpopulation of breast cancer cells, defined by a CD44+/ESA+/CD24lo cell surface phenotype, was capable of recapitulating the original tumour phenotype when transplanted into nonobese diabetic/severe combined immunodeficient (NOD/SCID) mice [48]. Since then, several groups have shown that breast tumours behave like an aberrant version of normal tissue and organise themselves into a cellular hierarchy with a cancer stem-like cell (CSC) at its apex. Indeed, expression of aldehyde dehydrogenase (ALDH), a stem/progenitor cell marker, has also been used to enrich for tumour-initiating CSCs [49]. The concept of CSCs has significant clinical implications, because these cells are believed to be responsible for tumour initiation and growth and are often resistant to chemo- and radiotherapy. In breast cancer, CD44+/ESA+/CD24lo cells are relatively insensitive to conventional chemotherapy and to radiation [50, 51].

Regarding endocrine therapy, there is accumulating evidence to suggest that an increase in breast CSCs occurs following endocrine therapy for ER*α* +ve tumours [52]. For example, Creighton and colleagues saw enrichment of cells with breast CSC features in tumour tissue derived from patients following therapy with an aromatase inhibitor (letrozole) [53]. Four other studies showed enrichment for CSC populations in ER*α* +ve breast cancer cell lines after tamoxifen treatment or oestrogen deprivation [18, 54–56]. The potential involvement of CSCs in breast cancer makes it imperative to further characterize these cells in order to find cellular signalling pathways that can be targeted to eradicate breast CSCs and, therefore, provide long-term disease-free survival.

One strong candidate pathway in this regard is the Notch pathway [21]. Pharmacologic and genetic inhibition of the Notch signalling can reduce breast CSC activity* in vitro* and tumour formation* in vivo*. Notch4 plays a particular key role in controlling breast CSCs [34]. In addition, it has also been reported that overexpression of specific Notch receptors is associated with treatment resistance in human breast cancer [57]. Likewise, it was shown that the use of antioestrogens can activate Notch signalling in breast cancer cells [17]. More recently Notch pathway was found to be hyperactivated in endocrine resistant breast cancer cells, and its inhibition blocked growth of these cells [58, 59]. Moreover, we recently demonstrated that increased JAG1-NOTCH4 signalling in human breast tumours is an important driver of cancer stem cells [56].

Besides Notch signalling, other CSC pathways are aberrantly activated in endocrine resistant cells. For example, Wnt and Hedgehog signalling pathways are active in CSCs and this has already been shown to promote tamoxifen resistance [55, 60]. Together these results suggest that inhibiting CSC signalling pathways will help to overcome endocrine therapy resistance and recurrence in ER*α* +ve breast cancer.

## 5. Conclusion

It is widely recognized that increased Notch signalling is one of the main drivers of cellular malignancies in breast cancer. Moreover, Notch pathway activation is commonly seen in response to targeted therapies. Therefore, combining current treatment options with a blockade of Notch signalling might be a feasible approach to consider. With this being said, individual Notch receptors are likely to regulate mammary epithelial and breast cancer cells in unique ways; hence it is vital to delineate the functional role for individual Notch receptors in mediating resistance to therapy in breast cancer.
